# Relationship of Vitamin A and Thyroid Function in Individuals With Obesity and After Laparoscopic Sleeve Gastrectomy

**DOI:** 10.3389/fnut.2022.824193

**Published:** 2022-03-15

**Authors:** Bingwei Ma, Peng Yang, Jingyang Gao, Lei Du, Chunjun Sheng, Taofeek Usman, Xingchun Wang, Shen Qu

**Affiliations:** ^1^Department of Endocrinology and Metabolism, Shanghai Tenth People's Hospital, School of Medicine, Tongji University, Shanghai, China; ^2^Department of Gastrointestinal Surgery, Shanghai Tenth People's Hospital, School of Medicine, Tongji University, Shanghai, China; ^3^Thyroid Research Center of Shanghai, Tongji University, Shanghai, China; ^4^Division of Endocrinology and Diabetes, Department of Pediatrics, Children's Hospital of Pittsburgh of University of Pittsburgh Medical Center (UPMC), University of Pittsburgh School of Medicine, Pittsburgh, PA, United States

**Keywords:** vitamin A, thyroid function, obesity, sleeve gastrectomy, subclinical hypothyroidism

## Abstract

Vitamin A deficiency (VAD) occurs in obesity and may be associated with thyroid dysfunction. We aimed to investigate the association of VA with thyroid function in obesity and after laparoscopic sleeve gastrectomy (LSG). Nine hundred and seventy-six obese subjects were enrolled for this study and were divided into VAD, marginal vitamin A deficiency (MVAD), and vitamin A normal (NVA) groups. VAD was defined as VA ≤ 200 ng/ml, MVAD was defined as VA > 200 but <300 ng/ml, and NVA was defined as VA ≥ 300 ng/ml. Thyroid function was compared among groups and the relationship of VA and thyroid function was analyzed. Two hundred and forty-four of the 976 obese subjects underwent LSG, and the change in thyroid function and VA at 3, 6, and 12 months after surgery was measured. Results showed that 37% of all the subjects had subclinical hypothyroidism (SH), and the SH group had lower VA levels than the non-SH group (*P* = 0.008). Forty-nine percent of all the subjects had MVAD, 9% had VAD, while the MVAD or VAD group had lower FT4 than the NVA group (*P* = 0.005 and *P* = 0.001). The VAD group also had higher TSH than NVA group (*P* = 0.037). VA was significantly negatively associated with TSH (*r* = −0.151, *P* = 0.006) and positively associated with FT4 (*r* = 0.228, *P* < 0.001). TSH was significantly decreased at 3, 6, and 12 months (3M: from 4.43 ± 2.70 to 2.63 ± 1.46 mU/l, *P* < 0.001; 6M: from 4.43 ± 2.70 to 3.84 ± 2.34 mU/l, *P* = 0.041; 12M: from 4.43 ± 2.70 to 2.85 ± 1.68 mU/l, *P* = 0.024). After LSG surgery, VA levels were slightly increased, when compared to pre-surgery levels, at 3, 6, and 12 months (3M: from 262.57 ± 68.19 to 410.33 ± 76.55 ng/ml, *P* = 0.065; 6M: from 262.57 ± 68.19 to 281.36 ± 93.23 ng/ml, *P* = 0.343; 12M: from 262.57 ± 68.19 to 300.37 ± 86.03 ng/ml, *P* = 0.083). SH group also had lower TSH and higher VA than the non-SH group at 3 months post-surgery [TSH: −1.4(−2.3, −0.3) vs. −0.2(−0.8, −0.2) mU/l, *P* < 0.001; VA: 163.99 ± 32.58 vs. 121.69 ± 27.59 ng/ml, *P* = 0.044]. In conclusion VA, which is related to thyroid hormone production, protects against thyroid dysfunction in obese subjects. The improvement of thyroid function in subjects with SH after LSG may be related to the increased VA levels observed post-surgery.

**Clinical Trial Registration:**

ClinicalTrial.gov ID: NCT04548232.

## Introduction

With changes in human lifestyle and environment, the prevalence of obesity is increasing as caloric intake in excess of caloric expenditure leads to a positive energy balance, obesogenic environment, and expression of genetic factors associated with poor nutrition (1). Obese individuals are predisposed to glucose and lipid disorders as well as vitamin deficiency (2). Most vitamins are deficient in obese individuals, especially the fat-soluble vitamins which include vitamin A (VA) (2). VA, also defined as all-trans-retinol and plasma VA levels, were significantly lower in patients with metabolic syndrome (MS) than those without MS (3, 4). VA has some influence on energy metabolism as it has been reported that rats fed a VA deficient diet exhibit increased adiposity and weight gain (5, 6).

Obesity has been significantly related to thyroid hormone dysfunction (7, 8). The metabolism of thyroid hormone requires iodine but is also influenced by micronutrients such as VA (9). VA regulates thyroid gland metabolism, the synthesis of thyroid hormone, the peripheral function of thyroid hormone as well as the secretion of thyroid stimulating hormone (TSH) by the pituitary (10, 11). Vitamin A deficiency (VAD) affects the synthesis of thyroglobulin, pairing of iodotyrosine residues to form T4 and T3 and reduces thyroid iodine uptake (10, 11), while VA supplementation reduced thyroid stimulation by thyrotropin and decreased the rate of goiter (12). However, no study focuses on the association of VA and thyroid dysfunction in obese individuals.

Laparoscopic sleeve gastrectomy (LSG) is one of the most effective methods to treat obesity as it not only led to decreased bodyweight but also improved metabolism (13–15). The effects of LSG on VA levels are inconsistent. One study indicated that VA levels were decreased after SG and gastric bypass (RYGB) (16). Another study pointed out that VAD was observed in 9.4% of SG and 15.9% of RYGB within 1-year post-operation and 5.2% of SG, and 7.7% of RYGB after 1 year (17). Another study showed no influence of bariatric surgery on serum VA levels (18). As to thyroid function, the consensus view is that LSG may improve thyroid function (19, 20).

Overall, the mechanism of VA on thyroid hormones remains unclear and few studies have investigated the association of VA and thyroid hormones in obesity. Therefore, we carried out a study to clarify the relationship between VA and thyroid hormones and the changes in VA and thyroid function after LSG.

## Materials and Methods

### Subjects

**Figure 1:**
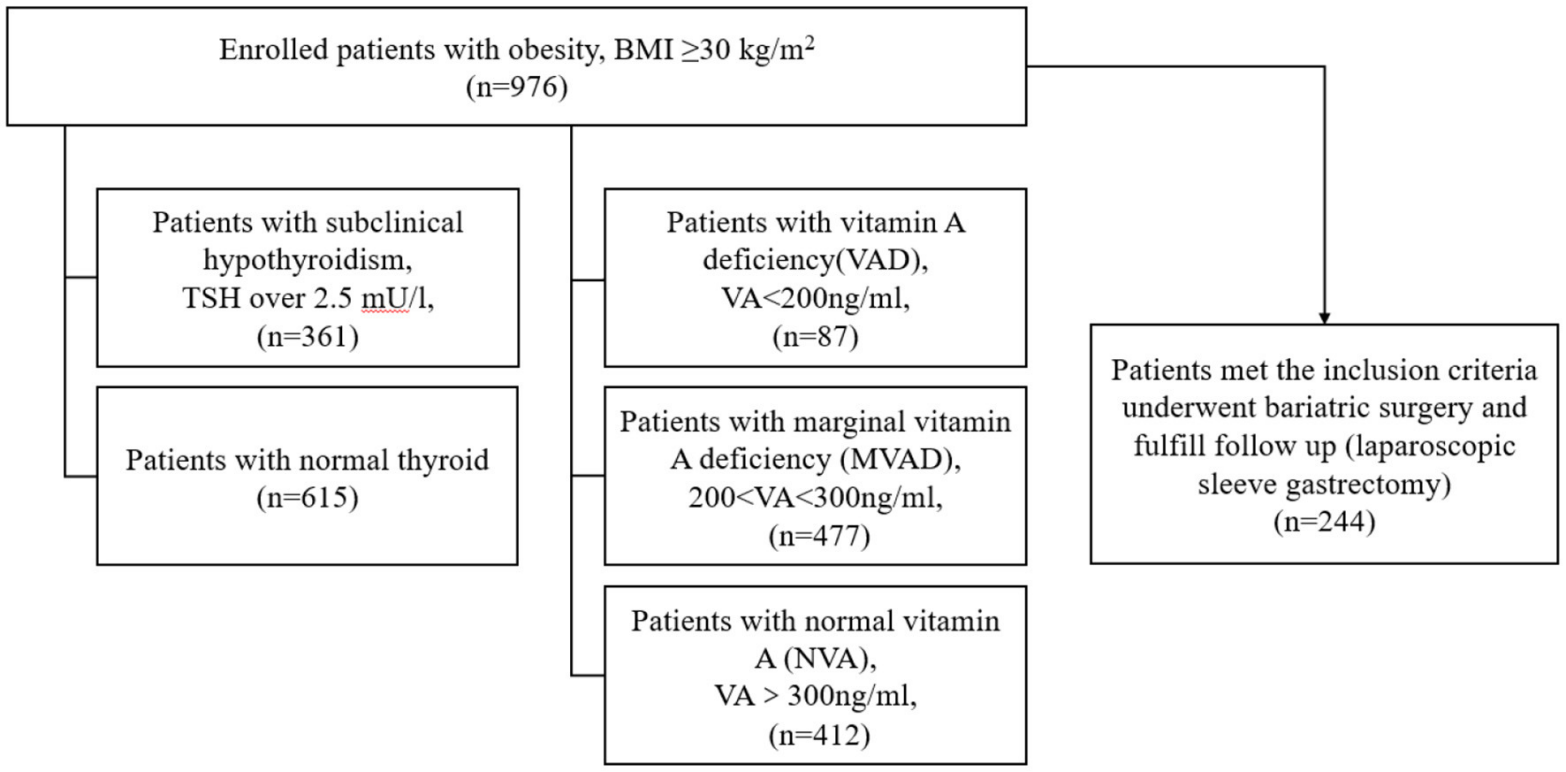
Flowchart of grouping and enrollment in this study.

This study enrolled 976 subjects with obesity. The definition of obesity is body mass index (BMI) ≥ 30 kg/m^2^ (21). They were divided into subclinical hypothyroidism (SH) group (TSH over 2.5 mU/l) and normal thyroid group depending on the TSH levels (8, 22, 23). VAD was defined as VA ≤ 200 ng/ml (24, 25), marginal vitamin A deficiency (MVAD) was defined as VA > 200 ng/ml but <300 ng/ml, and vitamin A normal (NVA) was defined as VA ≥ 300 ng/ml. Among them, 244 obese subjects underwent LSG. The inclusion criteria were as follows: (1) aged over 16 and <65 years old, (2) BMI ≥ 37.5 kg/m^2^, or BMI ≥ 37.5 kg/m^2^ complicated with type 2 diabetes (T2DM). The exclusion criteria including (1) secondary obesity including hypothalamus obesity, Cushing syndrome, etc., (2) Pregnant or lactating women, (3) contraindications of laparoscopic surgery, such as intra-abdominal infection and adhesion, gastrointestinal diseases, (4) serious heart, liver, and kidney failure which is intolerance to surgery. Assessments were taken at baseline and follow up measurements were taken at 3, 6, 12 months after surgery. The flowchart is as shown in Figure 1. All subjects enrolled did not receive VA supplement or any other intervention after surgery. All subjects enrolled were evaluated by an endocrinologist for thyroid function and vitamin A levels and assessed for compliance with enrollment criteria. The experimental scheme was approved by the Ethics Committee of local hospital and all subjects enrolled in this study signed the informed consent form.

### Measurements

Variables including age and gender were recorded. Anthropometric measurements including height and body weight were measured by professional staff and BMI was calculated by the following formula: body mass index (BMI) = body weight (kg)/height (m)^2^. Subjects were asked to fast for over 8 h before venous blood was collected among them. All the anthropometric and laboratory measurements were measured at baseline and follow up at 3, 6, 12 months after surgery. Laboratory measurements of glucose metabolism included fasting plasma glucose (FPG) and fasting insulin (FINS). FPG was measured by Roche Cobas c 701 fully automatic biochemical analyzer and FINS was measured by Roche Cobas e 601 analyzer. Homeostasis model assessment of insulin resistance (HOMA-IR) which was calculated to assess the insulin resistance by the following formula: FINS (uIU/ml) × FBG (mmol/l)/22.5 (26, 27). Lipid metabolic markers including total cholesterol (TCH), triglyceride (TG), high-density lipoprotein cholesterol (HDL-C), low-density lipoprotein cholesterol (LDL-C), and free fatty acid (FFA) were also measured with Roche Cobas c 701 fully automatic biochemical analyzer. Thyroid hormones including free triiodothyronine (FT3), free thyroxine (FT4), total thyroxine (TT3), total triiodothyronine (TT4), and TSH were tested to assess the thyroid function by ADVIA Centaur XP Immunoassay System. Serum vitamin A levels were determined using isotope dilution ultra-high-performance liquid chromatography-tandem mass spectrometry (ID-UPLC-MSMS).

### Statistical Analysis

Data of this study were statistically analyzed by SPSS software (Version 20.0). Normal distribution of data was also evaluated. If the continuous data were normally distributed, they were expressed as mean ± standard deviation (X ± SD). Otherwise, continuous data non-normally distributed was expressed as medians (interquartile ranges, IQR). Categorical variables were presented as numbers or percent. The normally distributed data were compared using an independent sample *t*-test, and non-normally distributed data were compared with the Mann & Whitney *U*-test. Pearson's or Spearman's test depended on the data normally distributed to investigate the correlations between thyroid hormone and other markers. A paired two-tailed *t*-test was adopted when comparing the data before and after surgery. A *P* < 0.05 was considered statistically significant.

## Results

### Comparison of Subjects With Different Thyroid Function and VA Levels

**Figure 2:**
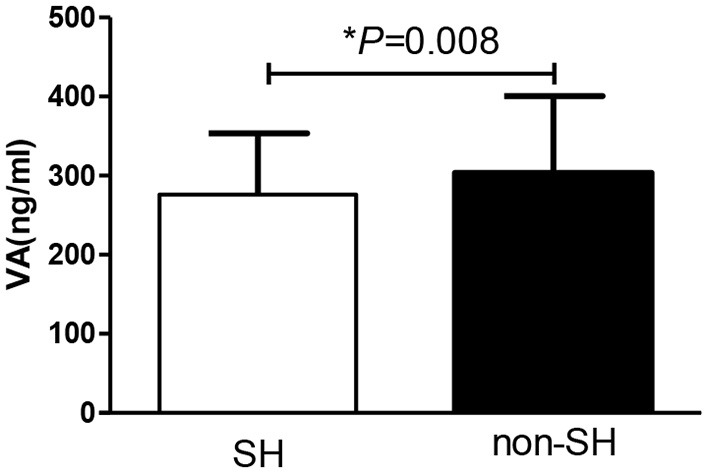
Comparison of VA levels between obese patients with or without SH.

**Figure 3:**
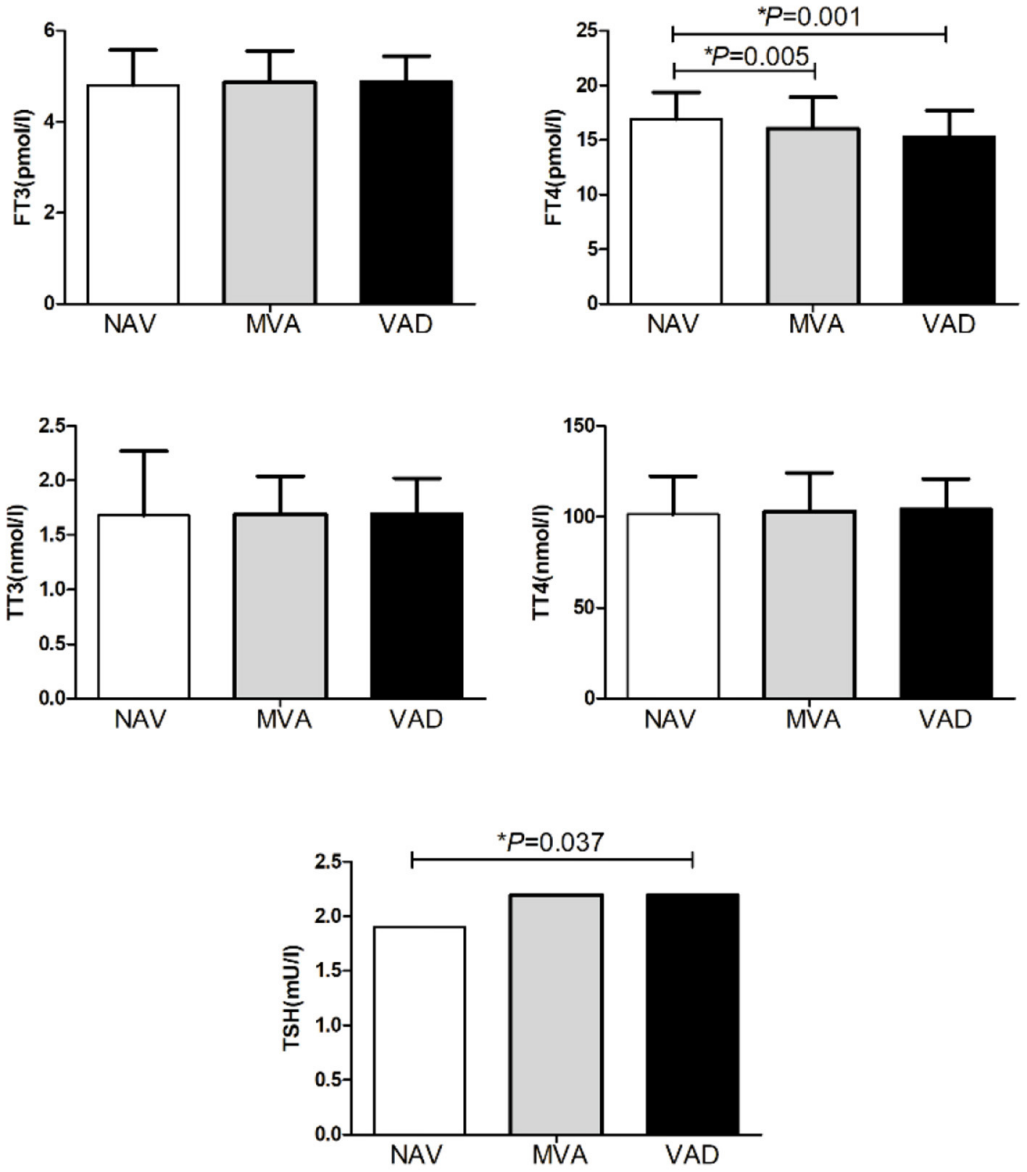
Comparison of thyroid hormone among obese patients with different degree of VA.

Thirty-seven percent of all participants in this study had SH and this group had lower VA than the non-SH participants (275.90 ± 77.71 vs. 303.37 ± 97.37 ng/ml, *P* = 0.008) as presented in Figure 2. Forty-nine percent of the participants had MVAD while 9% had VAD. The MVAD or VAD group of participants had lower FT4 than NVA group (*P* = 0.005 and *P* = 0.001). Meanwhile, VAD group had significantly higher TSH levels than NVA group [2.20 (1.85, 3.90) vs. 1.90 (1.20, 2.90) mU/l, *P* = 0.037; Figure 3]. Additionally, FPG, FINS, and HOMA-IR were significantly higher while HDL-C was significantly lower in the VAD group than the NVA group (*P* < 0.05; Table 1).

**Table 1:** Comparison of metabolism among patients with different degree of VA.

**Variables**	**NVA (VA ≥300 ng/ml) (*n* = 412)**	**MVA (200 < VA <300 ng/m) (*n* = 477)**	**VAD (VA ≤200 ng/ml) (*n* = 87)**
Age, years old	30.16 ± 12.34	31.62 ± 11.15	30.27 ± 6.15
Gender, male/female	218/194	161/316	27/60
Height, m	1.71 ± 0.08	1.68 ± 0.09	1.67 ± 0.06
Weight, kg	99.09 ± 26.00	99.93 ± 27.19	105.14 ± 28.08
BMI, kg/m^2^	33.91 ± 7.85	35.00 ± 7.80	36.81 ± 8.46
FPG, mmol/l	4.97 ± 1.04	5.21 ± 1.08	5.62 ± 1.00*
FINS, mU/L	12.05 (7.43, 24.24)	16.97 (9.03, 26.62)	19.30 (12.42, 33.45)*
HOMA-IR	2.61 (1.52, 6.35)	3.89 (1.91, 7.00)	4.60 (2.70, 8.83)*
TCH, mmol/l	4.39 ± 0.87	4.42 ± 0.82	4.34 ± 1.01
TG, mmol/l	2.63 ± 0.79	2.73 ± 0.78	2.59 ± 0.82
LDL-C, mmol/l	2.63 ± 0.79	2.73 ± 0.78	2.59 ± 0.82
HDL-C, mmol/l	1.13 ± 0.30	1.14 ± 0.45	0.99 ± 0.25*
FFA, mmol/l	0.57 ± 0.39	0.50 ± 0.20	0.47 ± 0.25

Continuous data are presented as means ± standard deviations (SD) or medians (interquartile ranges, IQR) based on the data distribution. Categorical variables are presented as number. ^*^Statistically significant (P <0.05). VA, vitamin A; BMI, body mass index; FPG, fasting plasma glucose; FINS, fasting insulin; HOMA-IR, homeostasis model assessment of insulin resistance; TCH, total cholesterol; TG, triglyceride; LDL-C, low-density lipoprotein; HDL, high-density lipoprotein; FFA, free fatty acid.

### Association of VA Levels and Thyroid Hormone

VA levels were significantly negatively associated with TSH (*r* = −0.151, *P* = 0.006) while positively associated with FT4 (*r* = 0.228, *P* < 0.001). Additionally, VA levels were significantly negatively associated with FPG (*r* = −0.188, P < 0.001) and positively associated with FFA (*r* = 0.124, *P* = 0.022). Adjusted for FFA and FPG, VA levels were still positively associated with FT4 (*r* = 0.150, *P* = 0.008; Table 2). Further regression analysis showed that FT4 was also significantly associated with VA levels (β = 18.238, *P* = 0.007) as in Table 3.

**Table 2:** Association of VA and thyroid function.

**Variables**	**All subjects (*n* = 976)**	**Adjusted for FFA and FPG**
	***r* (*P*)**	***r* (*P*)**
Age	NS	NS
Height	NS	NS
Weight	NS	NS
BMI	NS	NS
FPG	−0.188 (<0.001)	NS
FINS	NS	NS
TCH	NS	NS
TG	NS	NS
HDL-C	NS	NS
LDL-C	NS	NS
FFA	0.124 (0.022)	NS
FT3	NS	NS
FT4	0.228 (<0.001)	0.150 (0.008)
TT3	NS	NS
TT4	NS	NS
TSH	−0.151 (0.006)	−0.035 (0.532)

*Statistically significant (P < 0.05); NS, no significant; VA, vitamin A; BMI, body mass index; FPG, fasting plasma glucose; FINS, fasting insulin; HOMA-IR, homeostasis model assessment of insulin resistance; TCH, total cholesterol; TG, triglyceride; LDL-C, low-density lipoprotein; HDL, high-density lipoprotein; FFA, free fatty acid; FT3, free triiodothyronine; FT4, free thyroxine; TT3, total thyroxine; TT4, total triiodothyronine; TSH, thyroid stimulating hormone*.

**Table 3:** Regression analysis of VA and thyroid function.

**Variables**	**β**	**95%CI**	***P*-value**
TSH	−1.347	(−4.469, 1.776)	0.397
FT4	18.238	(4.932, 31.544)	0.007
FFA	37.718	(−20.038, 83.472)	0.229

*FFA, free fatty acid; TT4, total triiodothyronine; TSH, thyroid stimulating hormone; CI, confidence interval*.

### Change in Thyroid Hormone and Metabolism After LSG

**Figure 4:**
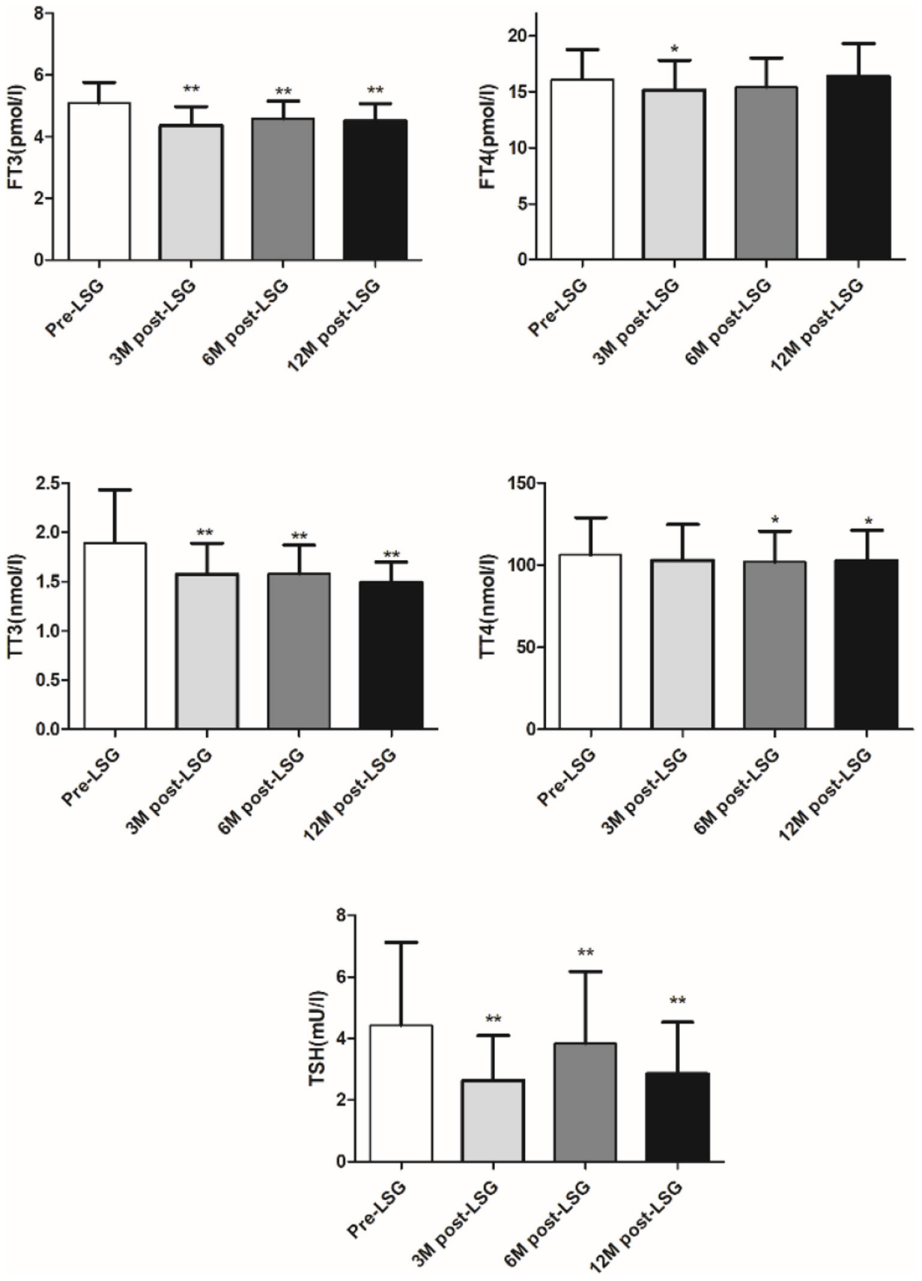
Change in thyroid hormone after LSG. *P < 0.05, **P < 0.001.

LSG led to significantly decreased body weight and improved glucose-lipid metabolism at 3, 6, and 12 months after surgery (*P* < 0.05; Table 4). Additionally, thyroid function was improved as TSH, FT3, and TT3 was significantly decreased at 3, 6, and 12 months after surgery (all *P* < 0.05). FT4 at 3 months, and TT4 at 6 and 12 months were significantly decreased after surgery as shown in Figure 4. Additionally, change in TSH at 6 months was positively associated with the change in BMI (*r* = 0.284, *P* = 0.026).

**Table 4:** Change in metabolic marker follow up 3, 6 month and 12 months post-operation.

**Variables**	**Pre-surgery**	**3M post-LSG**	**6M post-LSG**	**12M post-LSG**
Weight, kg	119.05 ± 22.43	94.76 ± 18.44**	93.37 ± 20.85**	81.34 ± 16.77**
BMI, kg/m^2^	41.74 ± 5.75	33.16 ± 4.73**	31.53 ± 5.19**	28.64 ± 4.50**
FPG, mmol/l	7.05 ± 2.38	4.53 ± 0.56**	4.49 ± 0.44**	4.53 ± 0.68**
FINS, mU/L	31.3 (22.25, 46.78)	10.2 (7.39, 15.40)**	9.27 (6.81, 14.25)**	6.96 (5.00, 10.10)**
TCH, mmol/l	4.50 ± 0.84	4.43 ± 0.76	4.41 ± 0.85	4.19 ± 0.85**
TG, mmol/l	1.85 ± 1.23	1.21 ± 0.39**	1.00 ± 0.34**	0.82 ± 0.28**
LDL-C, mmol/l	2.76 ± 0.78	2.83 ± 0.65	2.92 ± 1.22	2.53 ± 0.75*
HDL-C, mmol/l	1.05 ± 0.52	0.99 ± 0.22	1.17 ± 0.33	1.27 ± 0.28**
FFA, mmol/l	0.53 ± 0.20	0.58 ± 0.20	0.47 ± 0.19	0.53 ± 0.47

Continuous data are presented as means ± standard deviations (SD) or medians (interquartile ranges, IQR) based on the data distribution. *Statistically significant (P < 0.05); **Statistically significant (P < 0.001). VA, vitamin A; BMI, body mass index; FPG, fasting plasma glucose; FINS, fasting insulin; TCH, total cholesterol; TG, triglyceride; LDL-C, low-density lipoprotein; HDL, high-density lipoprotein; FFA, free fatty acid.

### Change in VA After Surgery

**Figure 5:**
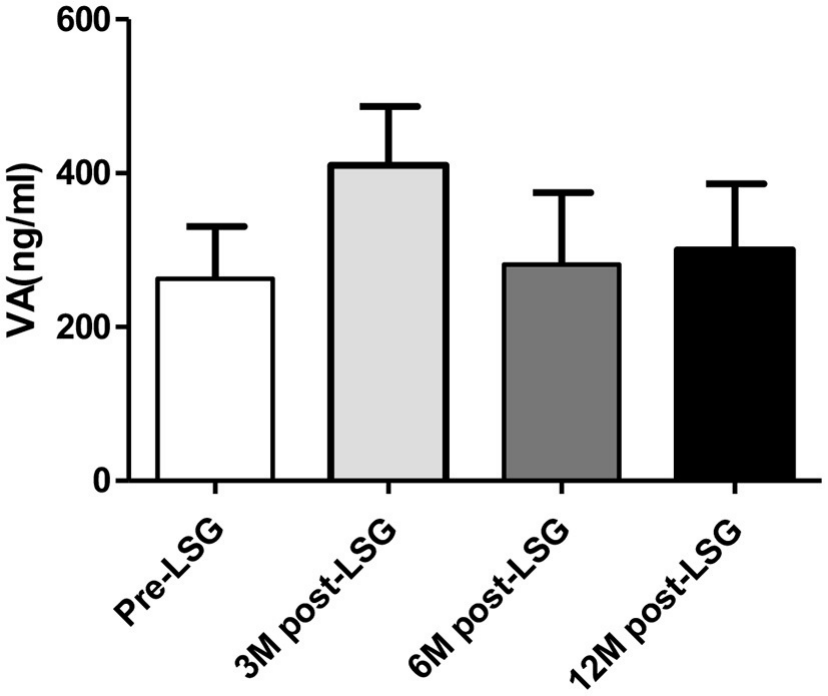
Change in VA levels after LSG.

**Figure 6:**
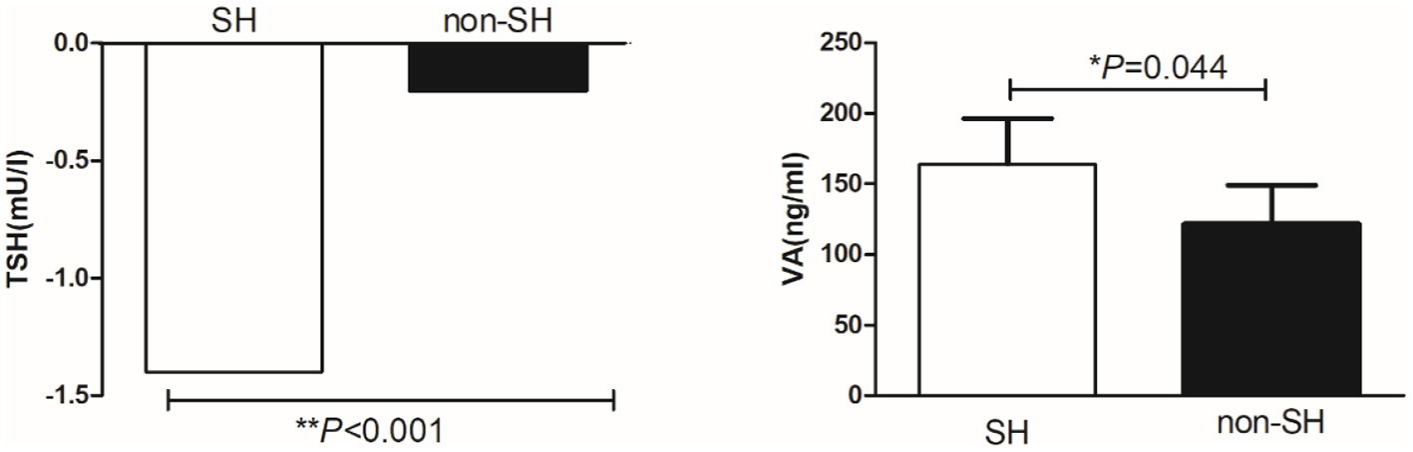
Comparison of change in TSH and VA between obese patients with or without SH.

VA levels were slightly increased after LSG at 3, 6, and 12 months (3M: from 262.57 ± 68.19 to 410.33 ± 76.55 ng/ml, *P* = 0.065; 6M: from 262.57 ± 68.19 to 281.36 ± 93.23 ng/ml, *P* = 0.343; 12M: from 262.57 ± 68.19 to 300.37 ± 86.03 ng/ml, *P* = 0.083) but these changes were not statistically significant as presented in Figure 5. The SH group had a lower TSH and higher VA than the non-SH group at 3 months [TSH: −1.4 (−2.3, −0.3) vs. −0.2(−0.8, −0.2) mU/l, *P* < 0.001; VA: 163.99 ± 121.69 vs. 32.58 ± 27.59 ng/ml, *P* = 0.044] as presented in Figure 6.

## Discussion

Obesity affects 27.5% of adults and 47.1% of children worldwide (28). Comorbidities associated with obesity include metabolic disorders as well as thyroid dysfunction and vitamin deficiency (2, 29). VAD affects over 30% of the global population, as well as the obese (10). VAD among school-age children in Chongqing, China was found to be significantly associated with obesity (30). VAD may also aggravate the pathological state of obesity (2). In our study, 37% of the enrolled subjects with obesity had SH. MVAD occurred in 49% of the included subjects with obesity and VAD occurred in 9% of the included subjects with obesity. However, few studies investigated the association of VA and thyroid function in an obese population. Therefore, we inferred that VA and thyroid disorders, which are common in obese subjects, may crosstalk with each other and that formed the basis of this study.

Synthesis and metabolism of thyroid hormones involve iodine, enzymes, and proteins and are also influenced by micronutrients and vitamins (9). VA takes part in the uptake of iodine while its deficiency impairs the synthesis of thyroglobulin and reduces the uptake of thyroidal iodine (31, 32). Animals studies also found that thyroid of VA deficient rats took up less radioiodide than thyroid of control rats (32). Thyroid hormone synthesis was decreased in the VA-deficient rats (11), and the thyroid weight of VA deficient rats was increased and the level serum thyroxine levels of VA deficient rats was decreased to one-half that of the control rats (11). Our study found that obese subjects with SH had lower VA than obese subjects with normal thyroid function. Obese subjects with MVAD or VAD had lower FT4 than obese subjects with NVA. Additionally, obese subjects with VAD had higher TSH levels than obese subjects with NVA. VA levels were significantly negatively associated with TSH while positively associated with FT4. Logistic regression analysis of risk factors for SH found that VA was a protective factor for SH. We inferred that VA and thyroid function interacted with each other in subjects with obesity and adequate VA may reduce the risk for hypothyroidism in obese subjects.

Bariatric surgery is one of the most effective methods to reduce body weight as well as improve metabolism (13–15). However, findings regarding change in VA after bariatric surgery has been inconclusive. Decreased VA micronutrient was observed after bariatric surgery including SG and RYGB (16). One study pointed out that VAD was uncommon preoperatively (2.7% SG vs. 1.7% RYGB) but increased after surgery (9.4% SG vs. 15.9% RYGB within 1 year post-operation, and 5.2% SG vs. 7.7% RYGB after 1 year) (17). However, another study found that there was no influence of bariatric surgery on serum VA levels (18). Our study found that VA levels were not decreased at 3, 6, and 12 months after LSG but were slightly increased. We inferred it may due to the ethnic differences and the choice of different types of operations. As we know, LSG led to a smaller risk for nutritional deficiencies while gastric bypass procedures are related to increased nutritional deficiencies because this kind of procedure is more complex which changes the gastrointestinal anatomy (33). On another hand, elevated C-reactive protein (CRP) concentration was associated with lower VA concentrations in morbidly obese subjects (34). LSG has effects on reducing body weight, improving metabolism and chronic low-grade inflammation with decreasing CRP (35). Therefore, the effect of improved metabolism and reduced CRP caused by LSG on VA may have counteracted the effects of surgery itself. LSG may therefore have no adverse effect on VA levels in Chinese subjects with obesity according to our findings.

A systematic review and meta-analysis proved that TSH, FT3, and TT3 were decreased, with non-significant changes in TT4, FT4, and rT3 levels after bariatric surgery (19). Serve obesity may be characterized by a mild reversible central resistance to thyroid hormones. Thyroid hormone resistance caused by obesity may occur through a mechanism similar to that of insulin resistance which could explain the increase in TSH similar to that observed in hyperinsulinemia (20). In our study, thyroid function was improved significantly at 3, 6, and 12 months after surgery. Also, FT3, TT3, and TT4 were decreased significantly after surgery. These results may also indicate that LSG has effects on improving thyroid hormone resistance in subjects with obesity although the underlying mechanism needs further experimental investigation.

Additionally, an animal study showed that VAD caused the reduction of the β-cell mass in the fetal pancreas and may contribute to glucose intolerance in adult rats (36). VA levels were compared in 191 subjects with MS and 98 subjects without MS, the results found that VA was significantly lower in subjects with MS than in healthy subjects (4). Also, the OR (95% confidence intervals) for MS is 0.942 (0.901–0.985) with a two-fold increase in total VA intake in women indicating decreases of 5.8% risk for MS (16). Our study showed that FPG, FINS, and HOMA-IR were significantly higher in VAD obese subjects than NVA obese subjects and VA levels were significantly negatively associated with FPG. VA may also have effects on lipid metabolism. HDL-C levels were found lower in subjects with VAD when compared to subjects with normal VA levels (4). VAD among school-age children in Chongqing, China has been reported to be significantly associated with hypertriglyceridemia (30). Our study also showed that HDL-C was significantly lower in VAD than in NVA subjects with obesity. Overall, VAD may also play a role in glucose-lipid metabolism but the underlying mechanism needs further exploration.

Our study clarified the association of VA and thyroid hormone in obese individuals before and after LSG. However, there also some limitations of our study. Firstly, the sample size is relatively smaller, and we did not compare the effects of different bariatric surgery on thyroid function and VA. Secondly, the follow-up time is relatively short, and we did not add the group with VA supplement. We will further expand the sample size, extend the follow-up time and add VA intervention to better understand the association of VA and thyroid hormone in obesity. Additionally, animal experiments can be undertaken to explore the underlying mechanism.

## Conclusion

VAD and SH are common in subjects with obesity. A crosstalk exists between VA and thyroid function as decreased VA in obese subjects was significantly related to thyroid dysfunction. Adequate VA levels may be a protective factor for thyroid function in obese subjects. Improved thyroid function was also observed after LSG and the improvement of thyroid function in obese subjects with subclinical hypothyroidism after LSG may be related to the increased VA levels observed in them.

## Data Availability Statement

The raw data supporting the conclusions of this article will be made available by the authors, without undue reservation.

## Ethics Statement

The studies involving human participants were reviewed and approved by Shanghai Tenth People's Hospital. The patients/participants provided their written informed consent to participate in this study.

## Author Contributions

SQ and XW made substantial contribution to the conception and design of the work. BM contributed to the data acquisition and draft manuscript. PY contributed to the analysis. JG contributed to the interpretation. LD helped in drafting the article. CS revised it critically for important intellectual content. TU revised the language. All authors gave the final approval of the version to be published and agreed for the accuracy or integrity of any part of the work.

## Funding

This research was supported by the Climbing Talent Program of Shanghai Tenth People's Hospital (2021SYPDRC047).

## Conflict of Interest

The authors declare that the research was conducted in the absence of any commercial or financial relationships that could be construed as a potential conflict of interest.

## Publisher's Note

All claims expressed in this article are solely those of the authors and do not necessarily represent those of their affiliated organizations, or those of the publisher, the editors and the reviewers. Any product that may be evaluated in this article, or claim that may be made by its manufacturer, is not guaranteed or endorsed by the publisher.
